# A Clinical Update on the Different Methods to Decrease the Occurrence of Missed Root Canals 

**DOI:** 10.7508/iej.2016.03.012

**Published:** 2016-05-01

**Authors:** Zahed Mohammadi, Saeed Asgary, Sousan Shalavi, Paul V. Abbott

**Affiliations:** a *Iranian Center for Endodontic Research, Research Institute of Dental Sciences, Shaheed Beheshti University of Medical Sciences, and Iranian National Elite Foundation, Tehran, Iran; *; b * Iranian Center for Endodontic Research, Research Institute of Dental Sciences, Dental School, Shahid Beheshti University of Medical Sciences, Tehran, Iran; *; c * Private Practitioner, Hamedan, Iran; *; d *School of Dentistry, University of Western Australia, Perth, Australia*

**Keywords:** Access Cavity, Cone-Beam Computed Tomography, Microscope, Missed Canals, Radiography, Root Canal Morphology, Transillumination, Ultrasonic

## Abstract

One of the main causes of endodontic treatment failure is the clinician’s inability to localize all the root canals. Due to the complex anatomy of the root canal system, missed canals are not uncommon. There are several strategies to decrease the possibility of missed root canals starting with good pre-operative radiographies. In order to overcome the limitations of conventional radiographies, cone-beam computed tomography (CBCT) can be considered. A correct access cavity preparation is of pivotal importance in localizing the orifices of the root canals. Furthermore, ultrasonics are very important devices to find missed canals. Increasing magnification and illumination enhance the possibility of finding all root canals during root canal treatment. The purpose of the present paper was to review all of the above techniques and devices.

## Introduction

Many of the problems that occur during root canal treatment (RCT) are because of inadequate knowledge about the tooth morphology and root canal system [1]. Thus, to ensure the long-term prognosis of a tooth undergoing RCT, it is imperative to assess the morphology of the root canals and their numerous variations before initiating RCT [2, 3].

***Modifying the access cavity***

The term *access cavity* refers to the part of the cavity from the occlusal table to the canal orifice. However, its design, which includes location, shape and size, depends on the position of the canal orifices as well as the position and curvature of the canal throughout its entire length. Sufficient tooth structure should be removed to allow instruments to be placed easily into the orifice of each canal without interfering with overhanging walls during root canal preparation. The orifice of each canal must be visible and easily accessible for instrument placement. If extra roots and/or canals are suspected at the diagnosis stage, the access cavity outline form should be modified [4].

In order to prepare a proper access cavity, the practitioner must have a thorough knowledge of the pulp chamber anatomy. The pre-operative radiographies should be carefully examined to determine the anatomy of the individual tooth that is about to be treated. The use of a specially-designed instrument such as a DG16 endodontic probe significantly facilitates the inspection of the pulp chamber floor and the discovery of canal orifices once the pulp chamber is exposed [5-7]. Clinical situations such as prosthetic crowns, large restorations, occlusal trauma and dystrophic calcification can alter the pulp chamber anatomy. 

The *anatomical laws* of Krasner and Rankow [5] highlight some useful general anatomical landmarks (independent of the crown anatomy) that may help the practitioner localize the orifices of canals that are not readily visible. Therefore, upon opening the pulp chamber, clinicians should consider these *laws* [5]. According to Krasner and Rankow [5], the floor of the pulp chamber is always located at the center of tooth at CEJ level.

Furthermore, pulp chamber walls are always concentric to the external surface of the tooth at CEJ level. As the above sentences reveal, the CEJ is the most consistent while repeatable landmark for locating the pulp chamber position. Furthermore, the color of the pulp chamber floor is always darker than the walls. They also demonstrated that except for the maxillary molars, the orifices of the canals are equidistant from a line drawn in a mesial distal direction through the pulp-chamber floor and the orifices of the canals lie on a line perpendicular to a line drawn in a mesial-distal direction across the center of pulp chamber floor [5]. Furthermore, Krasner and Rankow revealed that the canal orifices are always located at the angles of floor-wall junctions at the terminus of the root developmental fusion lines. 

As with all cavities and crown preparations, endodontic access cavities should be specifically designed for each tooth and for each patient. Parameters such as the degree of canal curvature, the position of the canal apex relative to its cusp tip, canal length, the degree of calcification, size and shape of the canals plus the position of the tooth in the jaw, all determine the specific design of an access cavity [4]. 

In maxillary anterior teeth, the prevalence of extra roots and extra canals is rare. Therefore, modification of the access cavity is not usually required [7]. In mandibular anterior teeth, however, the incidence of two canals is high [7]. Therefore, the access cavity should be extended in bucco-lingual direction to enable searching for two canals. Usually the labial canal is the first and the easiest canal to locate, so the clinician must carefully search for the lingual canal. 

Maxillary premolars usually have one and/or two canals and the access cavity should be oval-shaped with the longer diameter directed bucco-lingually so two-canals, if present, can be located [4]. Some maxillary premolars may have three canals (mesio-buccal, disto-buccal and palatal) so the buccal aspect of the pulp chamber should be carefully explored for this possibility since the anatomy will be similar to that of maxillary molars. Buccal canals in three-rooted premolars normally lie close to each other and are often covered by a projection of cervical dentin. Thus, the access opening should be modified to create a triangular conformation with the base of the triangle towards the buccal direction. The opening can be further modified if necessary at the bucco-proximal angles from the entrance of the buccal canals to the cavo-surface angles, resulting in a cavity with a T-shaped outline [8].

In mandibular premolars, the main canal orifice may split into two or three canals deep within the root. Thus, it is important to obtain good access to all canals. This may be achieved by using Gates-Glidden drills in a crown-down fashion. These drills should be withdrawn against the canal walls and away from the root concavities. This will reduce stress on the files used subsequently to shape the canals and minimize the risk of instrument fracture and canal transportation [9].

In maxillary molars, especially maxillary first molars, the presence of four canals are common. The mesio-buccal canal is often missed by clinicians. The location of its orifice varies greatly but it is generally located mesial to or directly on a line between the orifices of the mesio-buccal and palatal canals. As a general guide, it can usually be found within a zone located 3.5 mm palatally and 2 mm mesially from the mesio-buccal canal orifice. Therefore, because maxillary first molars almost always have four canals, the access cavity should have a rhomboid shape, with the corners corresponding to the four canal orifices. The access cavity should not extend into the mesial marginal ridge [10-13]. Although the mesio-palatal canal is often called the MB2 canal, this terminology is not anatomical and the more accurate and relevant term “mesio-palatal” should be used. The term is consistent with that used for all other anatomical structures, including all other root canals in teeth (for example, mesial canals of lower molars do not have the first and second mesial canals; they have mesio-buccal (MB) and mesio-lingual (ML) canals, lower incisors have a labial and lingual canal and not the first and second canals, *etc.*). The use of anatomical terms will help clinicians to understand and locate all canals. In lower molars, the chamber is usually triangular to square in shape. The access opening is triangular to slightly square on the occlusal surface, and its preparation should be distal to the mesial marginal ridge and primarily within the mesial half of the occlusal surface, keeping in mind that the distal extension of the access opening should extend into the distal half of the tooth. The access cavity for the mandibular first molar is typically trapezoid or rhomboid regardless of the number of canals present. When four or more canals are present, the corners of the trapezoid or rhombus should correspond to the positions of the main orifices. Mesially, the access does not need to invade the marginal ridge. Distal extension must allow straight-line access to the distal canal(s). The buccal wall forms a straight connection between the MB and DB orifices, and the lingual wall connects the ML and DL orifices without bowing [13-15].

Middle mesial (MM) canals are located between MB and ML canals of mandibular molars with the incidence of 1-15% for mandibular first molars and 10% for mandibular second molars. In almost all of the reported clinical cases, this canal joined the mesio-buccal or mesio-lingual canal in the apical third [16].

In mandibular second molars when three canals are present, the access cavity is very similar to that for the mandibular first molar or perhaps a bit more triangular and less rhomboid. The distal orifice is less often ribbon-shaped bucco-lingually; therefore, the buccal and lingual walls converge more aggressively distally to form a triangle. The second molar may have only two canals, one mesial and one distal, in which case the orifices are nearly equal in size and line up in the buccolingual center of the tooth. The access cavity for a two-canal second molar is rectangular, wide mesio-distally and narrow bucco-lingually. The access cavity for a single-canal mandibular second molar is oval and is lined up in the center of the occlusal surface [14, 15].

**Radiographic techniques to assist locating the canals**

***Conventional radiography***

Although periapical radiographies are two-dimensional images of the three-dimensional root canal system, their interpretation reveals external and internal anatomical details that suggest the presence of extra canals and/or roots [14]. For careful evaluation of the root canal morphology, at least two periapical radiographies should be taken using the parallel technique with either a mesial or distal horizontal tube shift [15]. The angled radiographies help to visualize the superimposed roots, allow good visualization of the buccal roots often covered by the palatal root of upper molars, displace the zygomatic process of the maxillary bone that can cover the apices of the molars, and suggest the position (buccal or lingual) of foreign bodies [15]. Martinez-Lozano *et al.* [17] examined the effect of x-ray tube inclination on the accuracy of determining the root canal anatomy of premolars. Their findings revealed that by varying the horizontal angle, the number of root canals observed in maxillary premolars coincided with the actual number of canals present. 

Pre-operative radiographies should be examined carefully; a sudden change in the radiographic density of the pulp space usually indicates an additional canal, whereas a sudden narrowing or even disappearance of the root canal space indicates a bi- or a tri-furcation [14]. Friedman *et al.* [18] showed the critical importance of pre-operative radiographies in identifying the complex canal morphology [18]. 

Post-operative radiographies can also provide valuable information about the presence and position of an extra root and/or canal. If the root filling material is not centered within the root, there may be a missed canal. Hoen and Pink [19] indicated a significant correlation between the asymmetric position of the previous root filling material and the ability to locate untreated root canals. 

Notwithstanding the above, radiographies may not reveal all canal bifurcations, accessory canals and apical deltas. Nattress *et al.* [20] reported that one-third of the canal bifurcations in roots assessed by viewing radiographies taken in the standard bucco-lingual direction were not visible. In another study, Bedford *et al.* [21] showed that the radiographies were not sensitive in assessing the number of the present root canals.

In summary, the information regarding root canal anatomy that is evident radiographically is valuable but also limited, and it should always be integrated with a careful clinical examination. Pre-operative radiographic analysis is critical for endodontics and multiple angled periapical views help to reveal the presence of roots and root canal systems. 

***Cone-beam computed tomography***

The amount of information gained from radiographies is limited by their two-dimensional nature [22, 23]. Other diagnostic methods such as computerized axial tomography (CT) can greatly facilitate assessment of the internal root canal morphology. One distinct advantage of CT scanning over conventional radiography is that it allows the operator to look at multiple slices of tooth roots and their root canal systems [22, 23]. CT images can also help to identify a greater number of morphologic variations than panoramic radiographies [24].

Cone-beam computed tomography (CBCT) is a medical imaging technique where the x-rays are divergent, forming a cone. During dental imaging, the CBCT scanner rotates around the patient's head, obtaining up to nearly 600 distinct images. A single 200 degree rotation over the region of interest acquires a volumetric data set. The scanning software collects the data and reconstructs it, producing what is termed a *digital volume* composed of three-dimensional voxels of anatomical data that can then be manipulated and visualized with specialized software [22-24]. 

CBCT scanning has been used in endodontics for the effective evaluation of the root canal morphology [25-28]. Matherne *et al.* [29] found that CBCT images always resulted in the identification of a greater number of root canals than digital radiographic images. Baratto Filho *et al.* [30] showed that an operating microscope and CBCT scanning were important for locating and identifying root canals, and CBCT scanning can be used for initial identification of the internal morphology of maxillary first molars. Llena *et al.* [31] showed that CBCT was useful to assess root canal morphology of mandibular premolars of a Spanish population. Cohenca and Shemesh [32] found that CBCT is a good option for identifying root canals and anatomical variations. Blattner *et al.* [33] as well as al-Shalabi *et al.* [34] revealed that CBCT scanning was a reliable method to detect the second mesio-buccal canal in human maxillary first molars. 

Moreover, the position paper published jointly by the American Association of Endodontists (AAE) and the American Academy of Oral and Maxillofacial Radiology (AAOMR) does not support the routine use of CBCT for all cases except when complex root canal anatomy is suspected [35]. This is supported by Reuben *et al*. [36] who reported that the modified canal staining and clearing technique was as accurate as CBCT in identifying root canal morphology. 

***Ultrasonic devices***

The use of ultrasonic devices in endodontics has enhanced the treatment and represents an important adjunct when managing difficult cases. The devices have become increasingly more useful in applications such as gaining access to orifices, cleaning and shaping, filling the canals, removal of intracanal materials and obstructions, and during endodontic surgery [37-39].

The use of ultrasonic tips with abrasive coatings helps remove dentine conservatively. The working end of these tips are typically about 10-times smaller than the smallest available round burs and consequently they can be used on the walls and/or floor of the pulp chamber to look for canal orifices. The use of such tips eliminates the bulky heads of conventional handpieces, which often obstruct vision, and they allow this *chasing* to be carried out under direct vision. Any use of instruments on the floor of the pulp chamber should only be carried out under direct vision because of the risk of perforation [39, 40]. 

Ultrasonic devices are particularly advantageous when attempting to locate the mesio-palatal canal in maxillary molars due to the cavitation effect [39]. Ultrasonic devices are used by some endodontists for this purpose although the majority of them appear to prefer the use of burs and endodontic explorers [39]. However, the use of ultrasonic tips may be more conservative. Alacam *et al.* [37] showed that the combined use of a microscope and ultrasonic devices increase the detection of mesio-palatal canals in maxillary first permanent molars. They showed that after locating the other three canals, the mesio-palatal canal was investigated in all teeth first without microscopy, then with the aid of a microscope and finally with the combined use of a microscope and ultrasonics. With these techniques, the mesio-palatal canal was detected in 62, 67 and 74% of the teeth, respectively. Stropko [41] revealed that when microscopy and ultrasonics were used, the rate of finding the mesio-palatal canals raised to 93%. Yoshioka *et al.* [42] also demonstrated that ultrasonic tips were effective in detecting the presence of mesio-palatal canals. 

***Microscopes***

For a long time, microscopes have been used in various medical specialties [43]. The reasons for their introduction to endodontics were the enhanced visibility and lighting. Carr and Murgel [44] stated that the microscope brings the practitioner right onto the pulp chamber floor and brings minute details into clear view. Lighting is significantly improved because the light of a microscope is parallel to the line of sight and will provide two to three times the light of a surgical headlamp [44].

The use of an operating microscope removes some of the guesswork that existed previously in many areas of endodontic treatment. Michealides [45] stated that the enhanced illumination and visibility enables endodontists to improve the predictability of their procedures. According to Khayat [46] one of the advantages of using a microscope is to increase the possibility of locating calcified and additional canals. Searching for calcified canals includes the use of the endodontic explorer, troughing with burs or ultrasonic tips and close visual inspection of the root anatomy, which gives clues to the location of the canals. One of the dangers in searching for calcified canals is the possibility of perforation. Using a microscope can give intimate detail of an area that otherwise would be under-illuminated and under-magnified, requiring guesswork and great caution [47, 48]. 

In a prospective clinical study, Sempira and Hartwell [49] indicated that use of a microscope did not increase the number of mesio-palatal canals located compared with those reports where access preparations were modified and the microscope was not used. Fogel *et al. *[50] examined the configuration of the location of the root canals in MB roots through a microscope, optical fiber and endodontic explorer. Their results showed that the accuracy in locating the mesio-palatal canal depended on the use of magnification, proper illumination and modified access. In an *in vitro *study on maxillary permanent molars, Kulild and Peters [51] demonstrated the presence of mesio-palatal canals in 96.1% of the teeth after sectioning 1 mm cuts, which were verified under microscopy. Ferguson *et al*. [52] showed that employment of a microscope considerably increased the chances of the location and endodontic treatment of mesio-palatal canals in maxillary molars. This finding was confirmed by Baldassari-Cruz *et al.* [12]. 

Coutinho-Filho *et al.* [53] showed that the adjunctive use of a microscope increased the ability of detecting the orifice of a mesio-palatal canal. Burhley *et al.* [47] utilized an *in vivo* clinical setting to evaluate if a microscope and/or dental loupes enhanced the ability of endodontists to locate mesio-palatal canals in maxillary molars. They found that those practitioners using the microscope located a mesio-palatal canal in 57.4% of the cases, while those using dental loupes found them in 55.3% of cases. When no magnification was used, a mesio-palatal canal was located only 18.2% of the time.

***Transillumination***

Transillumination is the technique of sample illumination by transmission of light through the sample. Transillumination is used in a variety of imaging methods. In medicine, transillumination generally refers to the transmission of light through tissues of the body. In dentistry, bright transilluminated light through the tooth can highlight dental caries, show signs of dental trauma such as coronal fractures and assist in locating the calcified canals. It has been suggested that transillumination of the cervical area of a heavily restored tooth can greatly improve visibility and reveal landmarks that otherwise remain invisible to the unaided eye. Transillumination can also aid in detecting calcified canals [54]. 

***Avoiding RCT in vital teeth***

Due to the complexity of root canal morphology, the reported success rates of RCT is lower than expected and had not improved over the last decades [55]. Preventive endodontics is a new concept for avoiding RCT and further treatment failure in teeth with vital pulps [56]. Successful techniques, which enable the clinician to avoid RCT failure, are the main priorities of Endodontics [57]; various vital pulp therapies have been developed as simple, safe, accessible, affordable, available and biologically-based treatments to address the issue [58-61].

***Other*** ***methods***

Some clinicians suggest staining the pulp chamber floor with 1% methylene blue dye. Methylene blue is a water-soluble dye that can be irrigated into a dry pulp chamber. The dye is absorbed into canal orifices and serves to visually map the hard-to-find canals [6, 62]. The use of this method requires care not to cause staining of the remaining tooth structure, especially in anterior teeth and premolars. Moreover, staining the grooves, line angles on floor of pulp chamber and *etc.* can impede locating the canal orifices. The so-called *sodium hypochlorite champagne bubble test* has been suggested as an aid to locating canal orifices. This method relies on the solution dissociating into Na^+^ and Cl^-^ ions which liberates free oxygen when placed in the access cavity. 

Gingival recession may reveal the root morphology and especially the presence of a furcation in some teeth and thus hint at the presence of two buccal roots. Probing the buccal sulcus to feel the root eminences and furcation anatomy may also help identify the presence of two buccal roots if present [7, 63]. Probing the palatal sulcus to feel the root eminences and furcation anatomy may also help to identify the presence of two palatal roots in maxillary molars [64]. 

Furthermore, Stropko [41] recommended the use of 17% EDTA and 95% ethanol to clean and dry the pulp chamber floor prior to visually inspecting the root canal system. In a tooth with a furcation, the orifices on the pulp chamber floor should be symmetrically positioned in relationship to each other and the external root surfaces.

## Conclusion

There are several strategies to decrease the possibility of missed root canals. Being familiar with these techniques and reaping the rewards of these special tests can be helpful in the success of endodontic treatment. 
